# Psychosocial Correlates of Depression and Anxiety in the United Arab Emirates During the COVID-19 Pandemic

**DOI:** 10.3389/fpsyt.2020.564172

**Published:** 2020-11-10

**Authors:** Justin Thomas, Mariapaola Barbato, Marina Verlinden, Carl Gaspar, Mona Moussa, Jihane Ghorayeb, Aaina Menon, Maria J. Figueiras, Teresa Arora, Richard P. Bentall

**Affiliations:** ^1^Department of Psychology, College of Natural and Health Sciences, Zayed University, Abu Dhabi, United Arab Emirates; ^2^Department of Psychology, College of Natural and Health Sciences, Zayed University, Dubai, United Arab Emirates; ^3^Clinical Psychology Unit, Department of Psychology, University of Sheffield, Sheffield, United Kingdom

**Keywords:** COVID-19, depression, anxiety, Arab, UAE, pandemic

## Abstract

The impact of the COVID-19 pandemic on mental health is likely to be significant. Identifying vulnerable groups during the pandemic is essential for targeting psychological support, and in preparation for any second wave or future pandemic. Vulnerable groups are likely to vary across different societies; therefore, research needs to be conducted at a national and international level. This online survey explored generalized anxiety and depression symptoms in a community sample of adults (*N* = 1,039) in the United Arab Emirates (UAE) between April 8th and April 22nd, 2020. Respondents completed symptom measures of depression (PHQ8) and generalized anxiety (GAD7), along with psychosocial and demographic variables that might potentially influence such symptoms. Bivariate and multivariate associations were calculated for the main study variables. Levels of anxiety and depression were notably higher than those reported in previous (pre-pandemic) national studies. Similar variables were statistically significantly associated with both depression and anxiety, most notably younger age, being female, having a history of mental health problems, self or loved ones testing positive for COVID-19, and having high levels of COVID-related anxiety and economic threat. Sections of the UAE population experienced relatively high levels of depression and anxiety symptoms during the early stages of the pandemic. Several COVID-related and psychosocial variables were associated with heightened symptomatology. Identifying such vulnerable groups can help inform the public mental health response to the current and future pandemics.

## Introduction

Severe Acute Respiratory Syndrome Coronavirus 2 (SARS-CoV-2) was first detected in Wuhan China in the latter part of 2019. The disease caused by this novel virus was officially named COVID-19 by the World Health Organization (WHO) on Feb 22nd, 2020 (1). After the virus spread internationally, the WHO officially declared COVID-19 a pandemic on March 11th, 2020 and many nations began acting to curb the spread. The United Arab Emirates (UAE), a federation of seven states on the east coast of the Arabian Peninsula, launched various infection prevention and control measures, which have included the promotion of social distancing, quarantine, the closing of educational and recreational facilities, the cessation of passenger air travel and curfews. Table 1, based on UAE governmental and media sources, provides a timeline of some of the primary infection prevention and control measures taken by the UAE Government.

**Table 1:** Timeline of the UAE's COVID-19 key infection prevention and control measures.

**Date announced**	**Effective date**	**Emirate**	**No. of cases**	**No. of deaths**	**Control and prevention measures**
Mar 3	Mar 8	UAE	6		All nurseries, schools and universities closed
Mar 12	Mar 14	Abu Dhabi	85		Remote working enacted for non-key workers
Mar 16	Mar 17	UAE	98		Places of worship closed
Mar 21	Mar 21	UAE	140	2	Closing of public recreational spaces (i.e., beach, parks)
Mar 22	Mar 22	Dubai			Social distancing in stores (1.5 m)
Mar 23	Mar 24	UAE	198		Closure of all shops and malls
	Mar 25	UAE			Grounding of flights
	Mar 26	UAE	333		3- days disinfection Program and Night curfew
	Mar 28	Abu Dhabi	468	4	First testing center opens
	Apr 5	Dubai	1,799	8	Movement permit requirements implemented
	Apr 7	Dubai	2,359	10	First drive through testing center open
	Apr 9	UAE	2,990	14	13 drive through testing centers open
	Apr 9	UAE			Mosques, churches, places of worship to stay closed as Ramadan approaches
	Apr 12	UAE		28	Home testing program for people of determination
Apr 15	Apr 16	Dubai	5,375		Field hospital with capacity to treat 3,000 COVID-19 patients opened
	Apr 28	Abu Dhabi	11,380		Mandatory COVD-19 test for mall employees before reopening Abu Dhabi malls

The UAE reported its first case on Jan 29th, 2020 (a family of four who had traveled to the UAE from Wuhan, China) and despite the extensive infection control efforts, the UAE confirmed its first two deaths on March 20th, 2020. New infections and deaths have ensued daily, and at the time of writing (May 18th, 2020) the UAE has tested more than 1 million people, reporting 23,358 cases (0.2% of the population) with 8,512 recoveries and 210 deaths. There is little doubt that the pandemic, and the necessary response to it, is causing considerable concern about its impact on mental health (2). From the grief associated with COVID-19 fatalities to the anxiety of testing positive, the pandemic has clear implications for mental health. Beyond the actual illness and the fear of contracting the virus, necessary restrictions placed on the freedom-of-movement, such as social/physical distancing, social isolation, quarantine and curfews, can also have negative implications for psychological well-being (2). Furthermore, anxiety surrounding the indirect effects of the economic implications associated with anti-pandemic measures, such as economic insecurity, may become an issue for some individuals and families.

Early research exploring the mental health consequences of COVID-19 in other nations support this view. A general population survey undertaken in the UK found that symptom measures of depression (PHQ9) and anxiety (GAD7) were elevated above those typically obtained in population surveys undertaken before the COVID-19 pandemic (3). A Chinese study, undertaken in Yunnan province, explored the rates of depression and anxiety among individuals affected or unaffected by quarantine measures, finding that those quarantined reported significantly higher rates of both (4). Other studies have looked at a variety of psychosocial factors correlated with mental health during the pandemic. For example, a study undertaken in Beirut, Lebanon, reported perceived social support as a potential resilience factor, that is, it was associated with lower levels of depression and anxiety symptomatology (5). Another study, this time in the USA, looked at religious coping among a Jewish community in New York, finding positive religious coping to be associated with less depressive symptomatology during the early stages of the pandemic (6). In another study, undertaken across, all 34 provinces of China, testing positive or having a relative who tested positive for COVID-19 was strongly associated with elevated depression and anxiety (7), All of these studies invariably reported levels of anxiety and depressive symptomatology elevated above pre-pandemic norms.

A rise in the symptoms of depression and anxiety is to be expected during times of international crisis. However, there is apprehension that this elevated symptomatology could translate into an increase in the number of clinically significant cases (2). There is evidence from earlier pandemics that this may be the case. A study undertaken in Hong Kong, for example, found that the Severe Acute Respiratory Syndrome (SARS) epidemic of 2003 was associated with a 30% increase in completed suicides among females aged 65 years and over (8). One year after the outbreak, survivors of SARS still reported elevated levels of anxiety and psychological distress (9). Another study following SARS survivors over 30 months described SARS as “a mental health catastrophe,” finding PTSD and depression to be the most prevalent long-term psychiatric morbidities (10). The work on SARS focused primarily on patients and healthcare workers. There is very little research exploring the broader mental health implications at the population level.

The possible rise in rates of depression and anxiety associated with the COVID-19 pandemic intersects with a point in time when several nations are already reporting a rising prevalence of depression and anxiety (11). Population-wide surveys of mental health in the UAE are relatively rare. However, based on data from the 2010 Global Burden of Disease study, the UAE has a rate of depression higher than the global average (12). Community surveys in the UAE (13–15) and neighboring Gulf states all confirm relatively high rates of depression and anxiety in the immediate region (16). How the current COVID-19 pandemic will impact mental health in the UAE and other nations remains an unanswered question. However, there is likely to be significant international variation, based on differing responses to the pandemic, variations in healthcare systems, as well as diverse demography and sociocultural norms. For example, citizens of the UAE frequently live in multi-generational extended family households (17). For many directly transmitted infectious diseases, household size plays a crucial role in transmission due to the greater strength of contacts between individuals sharing living arrangements (18). Household size may also impact levels of anxiety about possible infection. Similarly, relative to the global average, the UAE has a youthful population with estimates suggesting that one-half to one-third of the population are under the age of 25 years (19). Population age has been found to have a potential impact on epidemic patterns for infectious diseases, for example, in the absence of vaccination, population aging is shown to reduce per capita incidence of infection (20).

Here we report the initial findings from the first wave of a longitudinal, multi-wave survey of the psychological impacts of COVID-19 among a large community sample of residents and citizens of UAE, adapting survey methods used in a UK mental health survey (3). Administered during the early stages of the pandemic (April 8th to April 22nd, 2020), the study's primary aim is to identify psychologically vulnerable groups, by assessing the relationship between psychological well-being and variables likely to influence the symptoms of depression and anxiety. This includes variables related to living arrangements, COVID-19 infection status, and mental health history. The main variables explored in the study are detailed in Table 2.

**Table 2:** The study's main categorical variables with levels.

**Variables**	**Levels**	
Gender	Male	Female
UAE Citizen	Yes	No
College educated	Yes	No
Religious	Yes	No
Live alone	Yes	No
Children in household	Yes	No
History of mental health problems	Yes	No
Pre-existing health conditions	Yes	No
Perceived personal risk of COVID-19	High	Not High
COVID-19 infection status	Positive	Negative/Unknown
COVID-19 related anxiety	High	Not High
COVID-19 economic threat	High	Not High

*Age groupings are also included with four levels, 18–24, 25–34, 35–44, 45+ years*.

In light of the rising COVID-19 mortality and morbidity at the time of our study, and given the extensive, and necessary, restrictions imposed on freedom-of-movement, we anticipate higher levels of anxiety and depression compared to similar regional studies undertaken before the COVID-19 pandemic. More importantly, however, we also predict that COVID-related variables (e.g., infection status of self or loved ones) will be strongly associated with current levels of depression and anxiety. This study potentially contributes to what we know about the psychological consequences of pandemics in the UAE. The lack of knowledge about the impact of pandemics on population-level mental ill-health in the UAE, and elsewhere, is a critical gap to address. There is evidence from modeling studies that the psychological reactions (emotional and behavioral) to a pandemic can influence its course (21). Furthermore, the economic burden associated with elevated rates of mental ill-health are likely to negatively impact post-pandemic national recovery (3).

## Materials and Methods

### Participants

Participants (*N* = 1,039) were recruited via announcements in the UAE media and through the email networks of UAE's National Program for Happiness and Well-being. Participants were required to be residents of the UAE and aged 18 years or over. The sample was not representative of the whole UAE, but it did reflect many constituents. The sample comprised of people from 65 different nationalities, with citizens of the UAE (Emiratis) being the majority (73%). Reflecting the UAE's youthful population (19), the mean age was 28.33 (*SD* = 11.38) years. Females made up 85.6% of the sample, and the two most populous emirates/city-states represented, Abu Dhabi and Dubai, accounted for 59.2 and 31.7% of the sample, respectively.

### Measures

All demographic and COVID-related measures were translated and back translated by two bilingual (Arabic/English) psychologists. Additional measures such as the GAD7 and PHQ8 were already available in Arabic and English. Participants were presented with English and Arabic links affording them a choice as to which language they completed the survey in.

#### Demographic/Personal History Items

Demographic survey items were adapted, with permission, from those used in a similar UK study by Shevlin et al. (3). Example items included: “What is your highest qualification?” and “How many adults (18 years or above) live in your household (including yourself)?” Personal history items included questions about pre-existing health conditions and mental health history. For the current analysis all demographic and personal responses were assigned to binary categories. For example, college educated (yes/no), religious affiliation (yes/no), lives alone (yes/no) were all dichotomized. Age was assigned to four groupings as detailed in Table 3. The dichotomization and categorization of continuous variables is commonly used in health related research to stratify patients according to risk, and guide the allocation of resources based on easily communicated models of greatest need/highest risk (22).

**Table 3:** The frequency count and (percentage) for the study's main demographic variables.

**Variable**	**Frequency count and (%) per grouping category**					
Age Groups	*18–24*	*25–34*	*35–44*	*45+*		
	587 (56.5)	178 (17.1)	160 (15.4)	113 (10.9)		
Religion	*Muslim*	*Other*	*None*			
	789 (75.9)	168 (16.2)	80 (7.8)			
Education	*None*	*High Sch*.	*Bachelors*	*Masters*	*PhD*	
	6 (0.6)	420 (40.4)	385 (37.0)	161 (15.5)	68 (6.5)	
Employment	*Fulltime*	*Part-time*	*Self Emp*.	*Student*	*Unemp*.	*Other*
	308 (29.6)	32 (3.1)	32 (3.1)	496 (47.7)	89 (8.6)	82 (7.9)
Residence	*City*	*Rural*				
	939 (90.4)	100 (9.6)				
Occupancy	*Live alone*	*Two*	*Three*	*Four*	*Five*	*>5*
	83 (8.0)	162 (15.6)	127 (12.2)	127 (12.2)	133 (12.8)	423 (40.2)
Children	*None*	*One*	*Two*	*Three*	*Four*	*>4*
	227 (26.7)	167 (16.1)	209 (20.10)	127 (12.2)	97 (9.3)	154 (15.5)

*Children = number of children (under 18) living in the household, Occupancy = number of adults (18 years and older) living in the household*.

#### COVID-Related Items

The COVID-related items were also adapted from Shevlin et al. (3) These specifically probed infection status: “Have you been infected by the coronavirus COVID-19?” and “Has someone close to you (a family member or friend) been infected by the coronavirus COVID-19?” Response options were “yes,” “no,” and “unsure.” These two items were collapsed into a single binary item called COVID-19 Positive, where anyone who had tested positive or had a family member test positive was assigned a one/yes. Meanwhile, those who have tested negative or were unsure about COVID-19 status were assigned a zero/no. Additional items asked people to rate their level of worry about COVID-19 (“How anxious are you about the coronavirus COVID-19 pandemic? 0 = not at all anxious and 100 = extremely anxious”) and to indicate, from 1 to 10, their level of concern about finances: “On balance, how much are you worried about the way that your household finances have been affected by the coronavirus COVID-19 pandemic SO FAR?” These last two scales were represented by slider controls, where sliding the control past the 50% mark indicated an orientation toward the unpleasant side of the scale. These normally distributed items were also dichotomized for the current analysis, with respondents scoring over 50 categorized as high in COVID-related anxiety while those scoring 50 or below were categorized as low to moderate. The same dichotomization was applied to financial worry, with scores above five categorized as high in COVID-19 related economic threat, while those scoring 5 or less were classed as experiencing low to moderate economic threat/insecurity.

Participants were also asked to provide an estimation of their perceived personal risk of contracting COVID-19 in the coming month, this was a single item as used in an earlier UK-based study[4]. Response options were low, moderate and high. The low and moderate were collapsed into a single category, creating two categories of self-perceived risk, high and not high (low to moderate).

#### The Patient Health Questionnaire-8 (PHQ8)

The PHQ8 (23) is a widely used, standardized assessment of the prevalence and severity of depressive symptoms in the general population. It consists of eight questions probing the frequency of depressive symptoms over the past 2 weeks. Responses can range from 0 to 3 (0 = not at all, 1 = several days, 2 = more than half the days, 3 = nearly every day). Total scores, obtained by summing the responses to each item, range from 0 to 24. Total scores below 5 are viewed as indicating an absence of significant depressive symptoms. A cut-off score of ≥10 was used in the present study to indicate the presence of clinically significant depressive symptoms (moderate depression), this cut-off has previously been associated with excellent sensitivity and specificity for the detection of depressive disorders (23). The PHQ8 also uses scores of 15 and 20 and above to indicate severer levels of depression. The psychometric properties of the PHQ-8 scores have been widely supported, and the reliability of the scale among the current sample was excellent (α = 0.915).

#### The Generalized Anxiety Disorder-7 (GAD7)

The GAD7 (24) is a widely used measure of anxiety in the general population. Participants are asked to indicate how often, in the past 2 weeks, they have experienced each of seven main symptoms associated with generalized anxiety disorder. Total scores can range from 0 to 21 and are calculated by assigning scores of 0 (not at all), 1 (several days), 2 (more than half the days), and 3 (nearly every day), to item response. Scores of 5, 10, and 15 are considered cut-off points for mild, moderate and severe anxiety, respectively. The psychometric properties of the instrument have been widely supported (25), and the reliability of the scale among the current sample was excellent (α = 0.921).

### Procedure

The study received ethical clearance from the university's research ethics committee (R201213) and from the Ministry of Health and Prevention's research ethics committee (MOHAP/DXB-REC/ MMM/No. 49/2020). Data collection took place online (www.symplexsoftware.com/covid19/), where participants first selected their preferred language (63.2% selected English) and then read the participant information page, prior to consent giving. Consenting participants answered demographic and personal history questions first, followed by the PHQ8, the GAD7 and then the section specific to COVID-19 concerns. The median completion time for the survey was 18 min and 3 s.

## Results

### Descriptive Analysis

With the exception of age, all continuous variables were normally distributed. Age was left skewed due to a large number of relatively young people in the sample. Scores above the PHQ8 cut-off were notably high (58.4%). Similarly, scores above the GAD7 cut-off were also notably high (55.7%). The mean, standard deviation and bivariate correlation coefficient for all continuous variables are detailed in Table 4.

**Table 4:** Means and bivariate correlations for all continuous variables.

	***M***	***SD***	**PHQ8**	**GAD7**	**Economic threat**	**COVID-19 anxiety**	**Household occupancy**
Age	28.32	11.39	−0.337^**^	−0.251^**^	0.118^**^	0.056	−0.472^**^
PHQ8	12.10	7.60	–	0.757^*^	0.124^**^	0.181^**^	0.212^**^
GAD7	11.37	7.43		–	0.180^**^	0.314^**^	0.139^**^
Economic threat	6.42	3.37			–	0.455^**^	−0.065^*^
COVID-19 anxiety	33.58	33.15				–	0.014
Household occupancy	7.43	4.54					–

*Two-tailed bivariate Pearson's correlation, N = 1,039 to 1,024 due to occasional missing age data*.

* *p < 0.05*,

** *p < 0.001*.

### Regression Analysis

**Figure 1:**
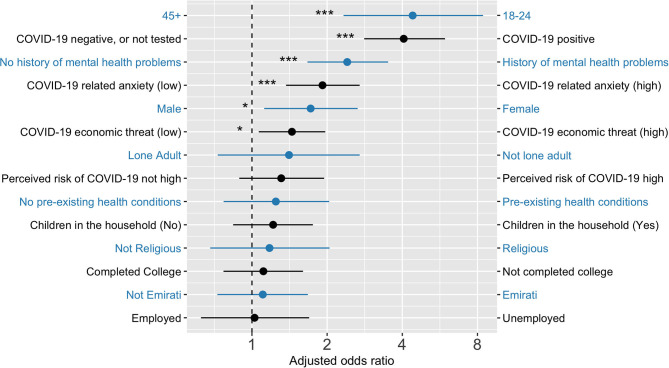
Adjusted odd ratios from a logistic regression predicting PHQ8 depressive symptom scores. Note: 18–24 years age group had significantly higher odds of depression and anxiety when compared to all other ag groups. Figure built using https://github.com/SourCherries/odds-forest. *p < 0.05, ***p < 0.001.

**Figure 2:**
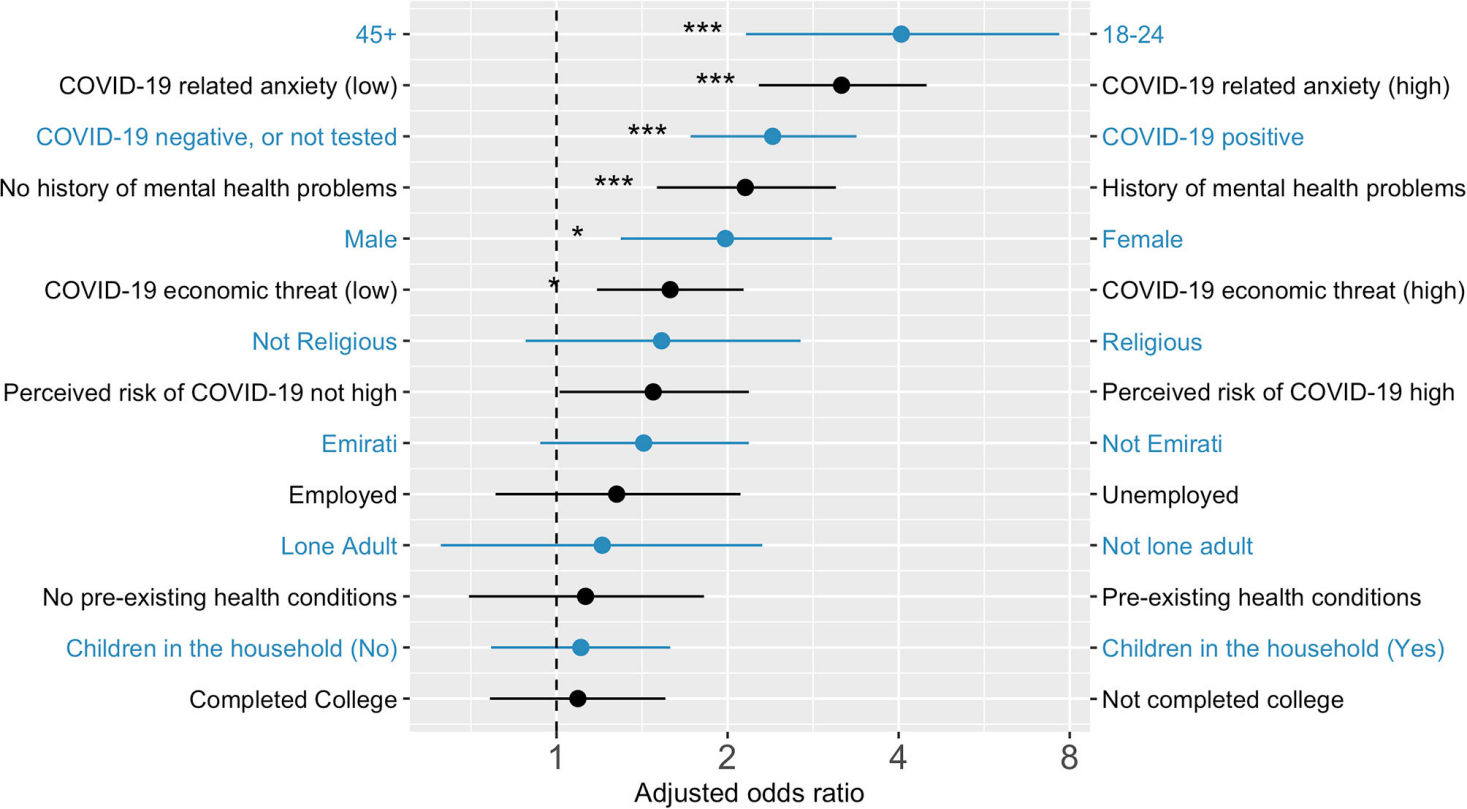
Adjusted odd ratios from a logistic regression predicting GAD7 anxiety symptom scores. Note: 18–24 years age group had significantly higher odds of depression and anxiety when compared to all other ag groups. Figure built using https://github.com/SourCherries/odds-forest. *p < 0.05, ***p < 0.001.

Bivariate and multiple logistic regressions were done with R (26), using generalized linear models in the base package. Two binary logistic regression models were used to predict caseness on Anxiety (GAD7) and Depression (PHQ8), computing bivariate odds ratios (OR) and multivariate adjusted odds ratios (AOR) for all predictor variables. The predictor variables were age, gender, education, employment status, citizenship, lone adult, number of children in household, pre-existing health condition, mental health history, COVID-19 infection status (self and other), and personal perceptions about risk of infection over the following month. The details of these analysis are detailed in Tables 5 and 6 with adjusted odds ratios clearly illustrated in Figures 1 and 2.

**Table 5:** Bivariate (OR) and multivariate (AOR) logistic regression predicting PHQ8 depressive symptom scores above cut-off.

	***N***	**Above cut-off PHQ8 *N* (%)**	**Odds ratio**	**Adjusted odds ratio**
**Age (years)**				
18–24	587	407 (69%)	–	–
25–34	178	101 (56%)	0.472 (0.333–0.668)^***^	0.573 (0.365–0.899)^*^
35–44	160	63 (39.4%)	0.286 (0.195–0.418)^***^	0.301 (0.176–0.51)^***^
45+	113	36 (31.9%)	0.217 (0.136–0.342)^***^	0.227 (0.119–0.43)^***^
**Gender**				
Male	145	55 (37.97%)	–	–
Female	889	547 (61.5%)	2.63 (1.83–3.83)^***^	1.72 (1.12–2.65)^*^
**Nationality**				
Other	281	117 (41.6%)	–	–
Emirati	758	490 (64.6%)	2.55 (1.92–3.39)^***^	1.11 (0.727–1.67)
**Religious**				
No	80	35 (43.8%)	–	–
Yes	959	572 (59.6%)	2.004 (1.262–3.205)^**^	1.175 (0.680–2.044)
**Completed college**				
Yes	613	315 (51.4%)	–	–
No	426	292 (68.5%)	2.119 (1.623–2.270)^***^	1.111 (0.796–1.6)
**Unemployed**				
No	950	553 (58.2%)	–	–
Yes	89	54 (60.7%)	1.087 (0.699–1.709)	1.025 (0.625–1.695)
**Lone adult**				
Yes	64	27 (42.2%)	–	–
No	975	580 (59.5%)	1.996 (1.995 – 3.367)^**^	1.406 (0.880 – 2.703)
**Children at home**				
No	278	135 (48.6%)	–	–
Yes	761	472 (62.0%)	1.805 (1.362–2.398)^***^	1.215 (0.840–1.754)
**History of mental health**				
No	828	460 (55.6%)	–	–
Yes	211	147 (69.7%)	1.832 (1.319–2.564)^***^	2.410 (1.669–3.509)^***^
**Pre-existing health conditions**				
No	926	539 (58%)	–	–
Yes	110	68 (61.8%)	1.136 (0.746–1.754)	1.247 (0.769–2.037)
**Tested positive for COVID-19 (Self or Loved One)**				
No	764	607 (58.4%)	–	–
Yes	275	216 (78.5%)	4.00 (2.857–5.682)^***^	4.049 (2.817–5.917)^***^
**See self as high risk for COVID-19 in coming months**				
No	855	476 (55.7%)	–	–
Yes	184	131 (71.2%)	1.905 (1.348–2.725)^***^	1.311(0.893–1.946)
**COVID-19 economic threat**				
No	423	225 (53.2%)	–	–
Yes	602	374 (62.1%)	1.477 (1.142–1.908)^**^	1.445 (1.065–1.965)^*^
**COVID-19 related anxiety**				
No	718	389 (54.2%)	–	–
Yes	315	212 (67.3%)	1.838 (1.383–2.457)^***^	1.916 (1.370–2.703)^***^

*AOR model included all variables listed above*.

*** *< 0.001*,

** *< 0.01*,

* *< 0.05*.

**Table 6:** Bivariate (OR) and multivariate (AOR) logistic regression predicting GAD7 anxiety symptom scores above cut-off.

	***N***	**Above cut-off GAD7 *N* (%)**	**Odds ratio**	**Adjusted odds ratio**
**Age (years)**				
18–24	587	373 (63.5%)		
25–34	178	93 (52.2%)	0.513 (0.364–0.724)^***^	0.532 (0.339–0.83)^**^
35–44	160	74 (46.3%)	0.459 (0.316–0.664)^***^	0.378 (0.221–0.639)^***^
45+	113	39 (34.5%)	0.311 (0.197–0.483)^***^	0.247 (0.13–0.464)^***^
**Gender**				
Male	145	52 (35.9%)		
Female	889	522 (58.7%)	2.5 (1.74–3.63)^***^	1.98 (1.3–3.05)^**^
**Nationality**				
Other	281	117 (41.6%)		
Emirati	758	490 (64.6%)	1.56 (1.18–2.07)^**^	1.42 (0.936–2.18)
**Religious**				
No	80	35 (43.8%)		
Yes	959	572 (59.6%)	1.988 (1.252–3.205)^**^	1.531 (0.885–2.688)
**Completed college**				
Yes	613	315 (51.4%)		
No	426	292 (68.5%)	1.664 (1.285–2.160)^***^	1.091 (0.763–1.555)
**Unemployed**				
No	950	524 (55.2%)		
Yes	89	55(61.8%)	1.290 (0.826–2.033)	1.276 (0.781–2.105)
**Lone adult**				
No	64	27 (42.2%)		
Yes	975	580 (59.5%)	1.656 (0.990–2.786)^*^	1.203 (0.625–2.304)
**Children at home**				
No	278	135 (48.6%)		
Yes	761	472 (62.0%)	1.529 (1.156–2.024)^**^	1.104 (0.769–1.585)
**History of mental health**				
No	825	460 (55.6%)		
Yes	211	147 (69.7%)	1.838 (1.333–1.585)^***^	2.151 (1.502–3.106)^***^
**Pre-existing health conditions**				
No	929	539 (58%)		
Yes	110	68 (61.8%)	1.212 (0.8–1.859)	1.125(0.699–1.818)
**Tested positive for COVID-19 (Self or Loved One)**				
No	764	607 (58.4%)		
Yes	275	216 (78.5%)	2.342 (1.733–3.195)^***^	2.398 (1.721–3.367)^***^
**See self as high risk for COVID-19 in coming months**				
No	855	476 (55.7%)		
Yes	184	131 (71.2%)	2.037 (1.447–2.899)^***^	1.479 (1.012–2.179)^*^
**COVID-19 economic threat**				
No	423	194 (45.9%)		
Yes	602	375 (62.3%)	1.927 (1.493–2.494)^***^	1.585 (1.178–2.137)^**^
**COVID-19 related anxiety**				
No	718	342 (47.6%)		
Yes	315	231 (73.3%)	3.165 (2.358–4.292)^***^	3.175 (2.268–4.505)^***^

*AOR model included all variables listed above*.

*** *< 0.001*,

** *< 0.01*,

* *< 0.05*.

Similar variables were statistically significantly associated with both depression and anxiety, most notably younger age, being female, having a history of mental health problems, self or loved ones testing positive for COVID-19, and having high levels of COVID-related anxiety and economic threat.

## Discussion

To the best of our knowledge, there is no previous research exploring the psychosocial correlates of infectious illness pandemics on the population of the UAE, or the broader Arab world. Even globally, the literature on the potential mental health implications of previous pandemics is relatively scarce. There are a few studies, primarily from the Far East, which focused on the SARS (8–10) and the H1N1 (27) pandemics of the first decade of the present century. With the notable exception of work undertaken in Canada (28), these earlier studies generally reported elevated levels of psychopathology (anxiety, depression) during the respective outbreaks, with their primary focus being healthcare workers and those who survived the illnesses The present study, however, explored depression, anxiety and the psychosocial correlates among a general community sample during the early stages of the COVID-19 pandemic. This was during the month of April, shortly after the national response (curfews, social distancing, working from home and the closure of retail and recreational venues) had been enacted. The primary aim of the study was to identify psychosocial and specific COVID-related variables that were associated with elevated levels of depression and anxiety. Identifying such variables can potentially help target support to vulnerable groups. A secondary aim was to assess levels of depression and anxiety, with the expectation that, relative to earlier regional surveys, symptomatology would be elevated.

There were several statistically significant variables associated with elevated depressive symptoms (scores 10 and above on the PHQ8). After age group, the foremost correlate was having tested positive for COVID-19 or having a similarly infected close friend or relative (COVID-19 positive). This finding is similar to data reported from a general survey in the UK (3). Also, in line with the UK survey, was the observation that rates of depression and anxiety were highest among the youngest age group (18–24 years). This finding is particularly significant for the UAE, which has a relatively youthful population (19). Much of the economic burden associated with depression arises from the relatively early age of onset and it's typically chronic course, having younger individuals experience depression is particularly problematic from the health economics perspective. The observation of poorer mental health among youth in the UAE, and elsewhere, may reflect a matrix of despair about the future. From the climate crisis to the employment-related threat of artificial intelligence, these are concerns that may be experienced more acutely by people who expect to witness them within their own lifetimes. A further correlate of depression in the present study was a pre-existing history of mental health problems, as assessed by simple self-report item on the survey. It might be that the pandemic, and the necessary response to it, exacerbate pre-existing morbidities and perhaps contribute to relapse in the vulnerable. Common mental health conditions, such as depression, have a chronic course and relapse is common with a mean of four major lifetime episodes (29). Furthermore, stressful life events, particularly those that disrupt social rhythms (which is likely if people are confined to home), are often implicated in the onset and reoccurrence of mood disorders (30, 31). As has frequently been observed, female gender was associated with a higher risk of having elevated anxiety and depressive symptoms. Such gender differences are typically explained in terms of gender-role socialization processes that lead to females being more likely to adopt passive ruminative responses to negative moods (32, 33). These ruminative response styles appear to represent a cognitive vulnerability in the context of depression (34, 35). A previous, large-scale, community survey undertaken in the UAE also reported females as being at greater risk for depression compared to their male compatriots (14). Cultural norms relating to gender-role socialization in the UAE may also play a role in the elevated rates of depression and anxiety observed in the current study. The final two variables associated with depression were specific to the current pandemic: COVID-19 related anxiety and COVID-19 economic threat (financial worries). This finding accords with the extensive literature on the links between economic/financial insecurity and depression (36).

The factors associated with elevated generalized anxiety symptoms were similar to those correlated with depression, with the addition of perceived risk of contracting COVID-19 in the next month. Elevated perceived risk of contagion would fit with ideas that trait anxiety leads to heightened risk perceptions and estimates (37).

There are perhaps also insights to be gained from noting the variables that, at least in the UAE context, were not associated with elevated depression or anxiety; notably religion, and having children in the household. Religion has previously been found to be a protective factor against depression (38–40) but in the present study it was not. However, we only assessed religion as an identity (affiliation) rather than an individual's levels of religious commitment. Having children at home during the pandemic was associated with depression in a similar COVID-19 survey undertaken in the UK (3). Having children at home while working from home may prove stressful for some families. However, in the UAE, among citizens, it is not uncommon to find three generations of the same family living in one household along with extended family members (17). In the present study, the mean number of people (children and adults) per house, for Emirati citizens, was 8.84 (*SD* = 4.29), for non-citizens the mean was 3.64 (*SD* = 2.62). Larger households might reduce the stress of having children at home, through increased support with home-learning and social support in general. Surprisingly, however, household size was not associated with levels of COVID-19-related anxiety. Given that there is a higher risk of infectious disease transmission in larger households (18), this may reflect an area of focus for future health messaging in the context of infectious illness outbreaks in the UAE.

A secondary aim of the study was exploring changes in symptom levels (depression and anxiety) relative to previous, pre-pandemic, regional survey work. The majority of recently published studies, exploring depression and anxiety in the UAE, tend to focus on clinical populations. Alsaadi et al. (41), for example, explored depressive symptoms among multiple sclerosis patients from the UAE, reporting a prevalence of 17.6% for depression and 20% for anxiety. Similarly, Alsaadi et al. (42) report a prevalence of 26.9% for depression and 25% for anxiety among UAE patients diagnosed with epilepsy. Despite chronic health conditions being associated with elevated levels of depression (43), both of these reasonably contemporaneous studies reported lower rates of depression and anxiety than the current study; 58.4 and 55.7% for depression and anxiety, respectively. Similarly, in a non-clinical sample of 302 medical residents in the UAE, depression rates ranged from 6 to 33% depending on residents' medical specialty (44). However, it should be noted that web-based surveys can be prone to self-selection bias (the most anxious and depressed are keenest to take the survey). Furthermore, it should be noted that differences in methods of data collection and mental health assessment make formal, between-studies, comparisons difficult. However, the high rates in the present study are likely, at least in part, to be related to the COVID-19 pandemic and the subsequent infection prevention and control measures.

This study has several important limitations. Firstly, the sample was not representative of the entire UAE population. Notably absent were male workers in fields such as construction and other manual endeavors. Reaching this group was beyond the scope of the present study based on time constraints and the necessary restrictions placed on movement during April 2020. Another important limitation is the correlational nature of the study, rendering all causal and temporal inferences tentative at best. However, obtaining a preliminary understanding of the psychosocial factors associated with elevated levels of depression and anxiety among segments of the UAE population during the pandemic may help inform public mental health plans for current and future outbreaks of infectious illness. Furthermore, post-pandemic economic recovery is likely to be significantly impacted by societal levels of mental ill-health. Exploring potential risk and resilience factors associated with psychological well-being may also help inform broader strategies aimed at national economic recovery.

## Data Availability Statement

The datasets presented in this article are not readily available because the ethical approval for the study requires that only anonymized data may be shared on request with verified researchers. Requests to access the datasets should be directed to Prof. Justin Thomas, Justin.Thomas@zu.ac.ae.

## Ethics Statement

The study received ethical clearance from the research ethics committees of Zayed University (R201213) and the UAE Ministry of Health and Prevention (MOHAP/DXB-REC/MMM/No. 49/2020). All participants provided informed consent prior to undertaking the survey.

## Author Contributions

JT: study design, write up, and project management. MB and RB: study design and write up. MV and JG: write up. CG: analysis and data visualization. MM: translation and write up. AM: translation, survey development, and data acquisition. MF and TA: review and data acquisition. All authors provided approval for publication of the manuscript content and agree to be accountable for all aspects of the work in ensuring that questions related to the accuracy or integrity of any part of the work are appropriately investigated and resolved.

## Conflict of Interest

The authors declare that the research was conducted in the absence of any commercial or financial relationships that could be construed as a potential conflict of interest.
